# Mothering Here and Mothering There: International Migration and Postbirth Mental Health

**DOI:** 10.1155/2012/593413

**Published:** 2012-11-14

**Authors:** Stephanie S. Bouris, Lisa A. Merry, Amy Kebe, Anita J. Gagnon

**Affiliations:** ^1^Ingram School of Nursing, McGill University, Montreal, QC, Canada H3A 2A7; ^2^Université Sainte-Anne, Pointe-de-l'Église, NS, Canada BOW 1M0; ^3^Women's Health Mission, McGill University Health Centre, Montreal, QC, Canada H3H 2R9; ^4^Department of Obstetrics and Gynecology and the Ingram School of Nurisng, McGill University, Montreal, QC, Canada H3A 2A7

## Abstract

Over 125,000 women immigrate to Canada yearly—most in their childbearing years and many having given birth before immigrating. We sought to (1) examine the background characteristics and mental health profile of women separated from their children due to migration and subsequently giving birth in Canada (“dual-country (DC) mothers”) and (2) contrast these with those of “non-dual-country” migrant mothers. Of 514 multiparous migrant women giving birth, one-fifth (18%) reported being separated from their children due to migration. Over one-third of DC mothers were living in poverty (36.0% versus 18.6%, *P* = 0.001), and one in seven was experiencing household food insecurity (16.3% versus 7.6%, *P* = 0.01). Over one-third had no partner (40.2% versus 11.4%, *P* = 0.00), and nearly one-quarter reported no available support (23.1% versus 12.2%, *P* = 0.007). Over three-quarters were asylum seekers or refugees (83.7% versus 51%, *P* = 0.00). More DC than non-DC mothers had symptoms of postpartum depression (28.3% versus 18.6%, *P* = 0.04), symptoms of clinical depression (23.1% versus 13.5%, *P* = 0.02), and anxiety related to trauma (16.5% versus 9.4%, *P* = 0.04). Results suggest that identifying DC mothers is a rapid approach to enable clinicians to target a subgroup of women needing special attention.

## 1. Introduction

*“The mother left 2 children behind [and] does not know what has happened to them.” This is a nursing note on mother from Zambia living in Toronto.*

In 2010, there were an estimated 214 million migrants worldwide [1]; between 2006 and 2010, 1.27 million migrated to Canada [2]. More than 52% of the newcomers to Canada are women, and of these, 65% are in their childbearing years [2]. Over a five-year period in Canada from1996to2001, the fertility rate among immigrant women was3.1children per woman—twice the national fertility rate of 1.5children per woman [3, 4]. Canadian family reunification policies and practices, such as a narrow definition of family, lengthy application processing times, and financial requirements, can lead to prolonged separation periods between family members [5]. The psychological impact due to family separation is compounded by loss of social networks and support, traumatic experiences before, during, and after migration, and low economic status [6, 7]. 

The toll that migration status and acculturation adjustments have on women's health and well-being has given rise to several studies [8–10]. Women who are separated from their children due to migration report considerable emotional strain including anxiety, loss, and guilt, and they are at greater risk for depression [11, 12]. Migrant women who recently gave birth in a new country display greater risks of depression during pregnancy and are at higher risk for symptoms of postpartum depression (PPD), attributing their symptoms among other reasons to social isolation, feeling overwhelmed, and financial worries [8, 13, 14]. Migration class is associated with regulatory restrictions related to eligibility for health care services at provincial and federal levels [15]. Although research has looked at the impact on women who are separated from their children due to international migration [11, 12] and the health outcomes of women giving birth in a new country [16], to our knowledge, no studies have simultaneously examined the postbirth health of women who are separated from their children due to international migration and have subsequently given birth in a new country (“dual-country” (DC) mothers). 

Upon migrating, there may be a range of ways in which a woman nurtures her relationship with her child(ren) overseas such as financial responsibility, training for adult life, communication, and intimacy [6, 17]. The nature of this relationship is not the focus of the current study, instead we aim to describe the mental health risk profile of women separated from their children due to international migration and who have subsequently given birth in Canada. 

We hypothesized that DC mothers would have poorer mental health profiles than non-DC migrant mothers, and we posed the following research questions: (1) What are the background characteristics and mental health profile of DC mothers? and (2) how do their characteristics and mental health profile differ from non-DC mothers?

## 2. Materials and Methods

### 2.1. Study Population

This study is a secondary analysis of 514 multiparous migrant women who participated in the childbearing health and related service needs of newcomers study. Details of this study have been reported elsewhere [16]. This dataset was ideal for this purpose because it was created in the context of a Canadian prospective cohort study that aimed to examine the health and related service needs of over 1,500 women and their infants. To be included in the original study, women had to be Canadian-born or newly arrived migrants (i.e., ≤5 years) with a known migration status (refugees, asylum seekers, and nonrefugee immigrants), have no major hearing impairment or mental condition, and give birth to a live child who would go home with her. To be included in the current study, mothers also had to have been born outside of Canada, be multiparous, and have answered the question “did any of your children stay in the country you left?” Of these, 92 responded positively to being separated from a child due to international migration, characterising them as DC mothers and the remaining 422 as non-DC mothers. The study received approval from the relevant research ethics boards and women gave informed consent prior to entry into the study.

### 2.2. Data Collection

Data collection took place between February 2006 and December 2009 at six Montreal, one Vancouver, and five Toronto hospital birthing units. Mothers were recruited and administered a series of 16 questionnaires over three points of contact: in hospital by a research assistant and at two home visits by a registered nurse at 7–10 days and 4 months postpartum. The mothers' medical records were reviewed in-hospital and additional demographic and migration data were obtained through interviews [18]. The 4-month visit was important as the nurse had established a rapport with the mother to be able to ask intimate questions regarding abuse and mental health. In the current study, we examined mental health using the following indicators: posttraumatic stress disorder (PTSD) symptomatology, risk of PPD, and clinical depression and anxiety symptoms.

The interview schedule included the Edinburgh Postnatal Depression Scale (EPDS) [19], Harvard Trauma Questionnaire (HTQ) [20]—sections I and III, and Hopkins Symptom Checklist-25 (HSCL) [21], all of which have been widely translated and used in a number of studies among diverse cultural groups and validated against Diagnostic and Statistical Manual of American Psychiatric Association, IV Version (DSM IV) for clinical diagnoses of mental mood disorders [13, 22, 23]. PPD was measured by the 10-item self-report EPDS with a score ≥10 to be suggestive of minor/major PPD symptomatology [19]. The first section of the HTQ lists 16 items describing a range of exposures to violence or torture, and a concern is noted if a mother has witnessed or experienced at least one of the items [20]. The first 16 questions of section III of this questionnaire are scored and suggest symptoms of PTSD if ≥2.5 [20]. Symptoms of anxiety and depression are indicated if a woman scored ≥1.75 in parts I and II, respectively, of the HSCL [24]. Report of being “physically hurt” within the last year was assessed with the Abuse Assessment Screen [25]. Social isolation was defined as reporting *somewhat disagree* to *strongly disagree* to “having relatives or friends that will help me out even if I cannot pay them back” or “having someone who loves and cares about me” according to the Personal Resource Questionnaire [26]. All questionnaires were available in 13 languages (Arabic, Cantonese, English, French, Farsi/Persian, Mandarin, Punjabi, Russian, Serbo-Croatian, Somali, Spanish, Tamil, and Urdu) and were back-translated and assessed for cultural appropriateness [27]. 

### 2.3. Data Analysis

Bivariate analyses using chi-square and Fisher's exact tests were performed comparing DC with non-DC mothers to answer our main research questions. In exploratory analyses, sub-group comparisons of DC and non-DC mothers within the most precarious migrant groups (asylum seekers and refugees) were also conducted. Data were analysed using PASW Statistics 18. 

## 3. Results 

Of 514 multiparous migrant women giving birth, one-fifth (18%) reported being separated from their children due to international migration.  Table 1 presents their background characteristics and those of non-DC mothers. Two-thirds of DC mothers did not complete high school (65.9% versus 45.5%, *P* = 0.00), over one-third were living in poverty (36.0% versus 18.6%, *P* = 0.001), and one in seven was experiencing household food insecurity (16.3% versus 7.6%, *P* = 0.01). Over one-third had no partner (40.2% versus 11.4%, *P* = 0.00) and nearly one-quarter reported no available support (23.1% versus 12.2%, *P* = 0.01). Over three-quarters were asylum seekers or refugees (83.7% versus 51%, *P* = 0.000), and far less than half had their initial prenatal visit during the first trimester (42.2% versus 60.2%, *P* = 0.008). Although not statistically different from non-DC mothers, it is of note that one in seven (14.4%) DC mothers had no health insurance coverage, and nearly one-third (32.6%) gave birth by Caesarean section. In terms of other psychosocial variables, DC mothers were more likely to report physical abuse in the last year (14.8% versus 5.2%, *P* = 0.003) and having experienced or witnessed a traumatic event (72.2% versus 49.8%, *P* = 0.00).

Mental health outcomes for DC and non-DC mothers are reported in Table 2. Contrasted with non-DC mothers, DC mothers experienced more symptoms of PPD (28.3% versus 18.6%, *P* = 0.04), anxiety (16.5% versus 9.4%, *P* = 0.05), and clinical depression related to trauma (23.1% versus 13.5%, *P* = 0.02). Although it did not reach statistical significance, symptoms of PTSD were reported more frequently in DC mothers (7.7% versus 3.8%, *P* = 0.16). Comparisons of DC to non-DC mothers restricted to within the asylum-seeking/refugee group were not statistically different; however, rates of mental health outcomes tended toward greater risks to DC mothers for all outcomes. The highest was a 50% greater risk of PPD at 4 months (29.9% versus 20.0%, *P* = 0.08), followed by a 38% greater risk of PTSD (9.2% versus 5.7%, *P* = 0.29), and a 33% greater risk of clinical depression (26.3% versus 17.5%, *P* = 0.10).

## 4. Discussion 

Our study shows that the constellation of background characteristics which describe DC mothers, combined with their mental health profiles, suggest that they are an extremely high-risk group warranting case finding and additional support and care. Their mental health profile is poorer than other migrant mothers and is not explained by being in a precarious immigration class. The mental health issues they experience include symptoms of PPD risk for clinical depression and anxiety related to trauma. And these challenges are often experienced in the context of low formal education, low household income, food insecurity, single parenthood, lack of social support, limited time in Canada, limited host language ability, absence of health insurance, and having experienced a surgical birth.

Previous research has studied the impact of separation on the mother [11, 12], however to our knowledge, none has investigated the health or well-being of women who subsequently gave birth in a new country. The rates of harmful background psychosocial experiences and mental health outcomes of DC mothers we found in our study are greater than those found in studies of other populations. For example, as regards food insecurity, 4.1% of Canadian households with children have ever had to cut the size of meals or skipped meals [28], compared to the nearly four times greater rate of the DC mothers in our study. Canadian studies have estimated the prevalence of physical abuse during pregnancy in the general population to be about 6% [29, 30]. Our estimates for non-DC mothers are close to this rate (5.2%), while DC mothers were more than twice the rate (14.8%). 

DC mothers are at higher risk of PPD at 4-month postbirth than non-DC mothers (28.3% versus 18.6%), although both groups have higher rates than other studies which report an average prevalence of 13% [31]. Our findings that DC mothers exhibit symptoms over twice this rate may speak to the constellation of factors making up their background. DC mothers exhibited nearly twice the rate of signs of clinical depression than non-DC mothers (23.1% versus 13.5%) and more experienced anxiety (16.5% versus 9.4%). More of the DC mothers had past experiences of violence and trauma, and these past traumatic experiences could contribute to the observed higher rates of depression and anxiety in addition to separation from their children. Previous research has shown both that “…having children who lived elsewhere predicted the likelihood of major depression” [12] and that being with one's family can have a protective effect on trauma victims [7]. Anxiety is likely further exacerbated, since one or more of her children remain in the country where she experienced violence, and from where she has sought refuge. Reducing the lengthy immigration application processing time, financial requirements, and narrow definitions of family may reduce the long separation periods between family members. Finally, asylum-seeking women who give birth may further fear that their applications for asylum may be rejected while their infant is permitted to stay as a Canadian citizen, leaving them to be forced to choose to remain illegally in Canada, return with the infant to an unsafe environment, or leave the infant in Canada with other caregivers. 

Our study is not exempt from limitations. The relatively small number of DC mothers in our study prevented us from being able to perform multivariate analyses; however, analyses restricted to the combined asylum-seeking and refugee group did offer evidence that the difference in the two groups by immigration class was not responsible for the differences in mental health outcomes. The tools used for mental health risk do not constitute diagnostic tools on their own, and referral and followup with community mental health practitioners are warranted. 

Care providers working with migrant women in the perinatal period may wish to do screening to determine if the woman is (or is soon to be) a DC mother, knowing that she is more likely to experience poor mental health than non-DC mothers. Referral to mental health workers and relevant community agencies may be appropriate. Home visits and community activities may help to address isolation and minimize harmful mental health symptomatology. Assistance in reducing lengthy immigration application processing times through letter writing in support of a woman's case for family reunification could help to reduce the long separation periods between family members who could offer needed social support. This may be particularly beneficial for those who were victims of trauma since the literature shows that being with one's family can have a protective effect [7]. 

Research to further examine the causes of food insecurity such as isolation, inaccessibility to food programs (e.g., food banks), or insufficient income is warranted to develop targeted policies and interventions. Future research on how separation from children affects mothers' health and mental wellbeing with a subsequent birth in a new country is also needed.

## 5. Conclusions

The constellation of background characteristics which describe DC mothers, combined with their mental health profiles, suggests that they are an extremely high-risk group warranting case finding and additional support and care. Their mental health profile is poorer than other migrant mothers and is not explained by being in a precarious immigration class. Mental health issues include symptoms of PPD, risk for clinical depression, and anxiety related to trauma. These concerns were often present in the context of low formal education, low household income, food insecurity, lack of social support, limited time in Canada, limited host language ability, absence of health insurance, and having experienced a surgical birth. Our results suggest that identifying DC mothers is a rapid approach for obstetricians and gynecologists to identify a subgroup of migrant women who may be at greater mental health risk than other migrant mothers in their care. Additional support during the perinatal period and policies to promote rapid family reunification warrant consideration. 

## Figures and Tables

**Table 1:** Background characteristics for DC and non-DC mothers.

	Dual-country mothers *N* = 92	Non-dual-country mother *N* = 422	*P* value
Demographic			

Mean [SD] maternal age (yr)	32.0 [5.3]	31.0 [5.3]	.000
Education < 12 yrs, %	65.9^a^	45.4^b^	.000
Household income < $10,000 CAD/yr, %	36.0^c^	18.6^c^	.001
Not living with a male partner, %	40.2	11.4	.000

Migration			

Lived in Canada <2 years, %	52.2	50.0^d^	.706
Limited or no official language fluency (i.e., English or French) at 4-month home visit, %	32.6	40.3	.171
Mother's region of birth by UN world macroregion			
Africa, %	33.7	17.5	
Asia, %	20.7	38.9	
Europe and North America, %	2.2	8.1	.000
Latin America*, %	43.5	35.5	
Migration status at time of recruitment			
Asylum seeking, %	56.5	34.6	
Accepted refugee, %	27.2	16.4	.000
Nonrefugee/family class, %	16.3	49.1	
No health insurance at time of delivery, %	14.0^d^	9.6^e^	.177

Obstetrical			

First contact with health care provider during pregnancy			
First trimester, %	42.2^d^	60.2^f^	
Second trimester, %	47.8	33.8	.008
Third trimester, %	10.0	6.1	
Caesarean birth, %	32.6	39.6	.213

Psychosocial			

Mother feels that she has no one to help her and/or no one who cares about her at 1-week postpartum, %	23.1^a^	12.2^b^	.007
Mother reports physical abuse in the last year (measured by the Abuse Assessment Screen), %	14.8^g^	5.2^g^	.003
Mother reports experiencing or witnessing a traumatic event (measured by Harvard Trauma Questionnaire), %	72.2^d^	49.8^d^	.000
Mother cutting or skipping meals due to lack of resources 1-week postpartum, % 4-month postpartum, %	15.2 16.3	7.8 7.6	.026 .009

^a^Missing data on one woman.

^b^Missing data on three women.

^c^Missing data (*n* = 75 and *n* = 370) as mothers either did not know or did not want to disclose their household income.

^d^Missing data on two women.

^e^Missing data on seven women.

^f^Missing data on 28 women.

^g^The abuse questionnaire was only administered if no male was present in the household to ensure the mother's safety (*n* = 81 and *n* = 306).

*Includes the Caribbean, Central and South Americas.

**Table 2:** Mental health outcomes of DC and non-DC mothers.

	All migrants			Asylum-seeker/refugee subgroup		
	Dual-country mothers *N* = 92	Non-dual-country mothers *N* = 422	*P* value	Dual-country mothers *N* = 77	Non-dual-country mothers *N* = 215	*P* value
Risk for postpartum depression (PPD) (score ≥ 10 on the Edinburgh Postnatal depression scale)						
1-week postpartum, %	25.3^a^	23.4^b^	.710	26.3^a^	24.9^c^	.805
4-month postpartum, %	28.3	18.6	.036	29.9	20.0	.076
Anxiety related to trauma at 4 months (measured by Hopkins Symptom Checklist, part I), %	16.5^a^	9.4^d^	.046	18.4^a^	15.2^b^	.507
Clinical depression symptomatology (measured by Hopkins Symptom Checklist, part II), %	23.1^a^	13.5^d^	.021	26.3^a^	17.5^b^	.100
Posttraumatic stress disorder (PTSD) symptomology at 4 months (measured by Harvard Trauma Questionnaire), %	7.0^a^	3.8^e^	.157	9.2^a^	5.7^b^	.289

^a^Missing data on one woman.

^b^Missing data on four women.

^c^Missing data on two women.

^d^Missing data on six women.

^e^Missing data on five women.
